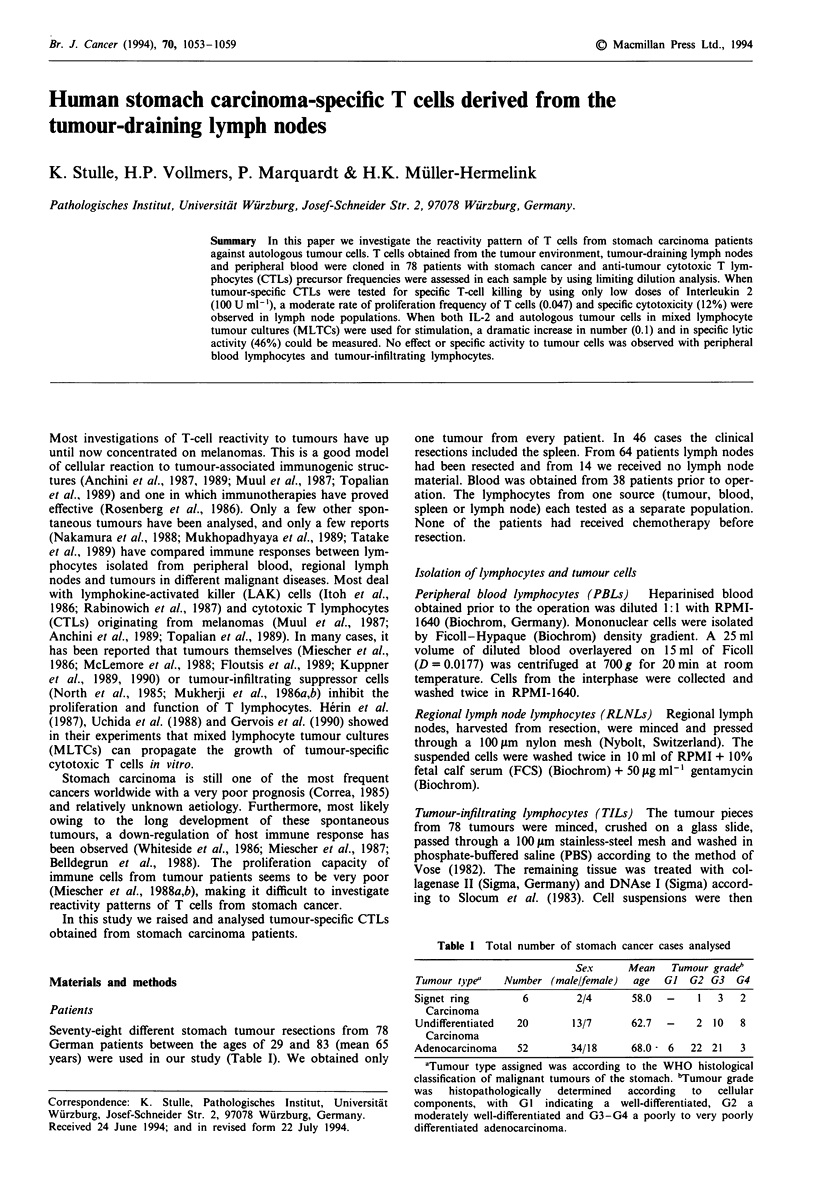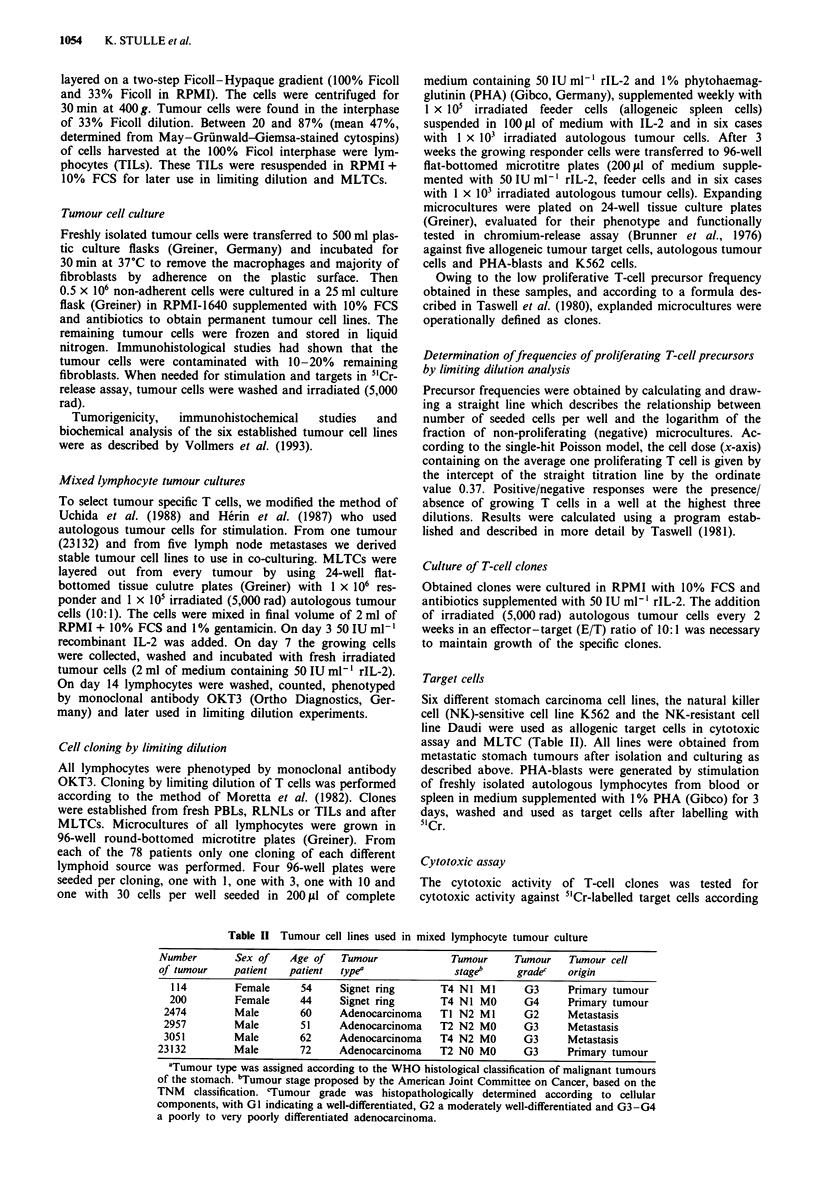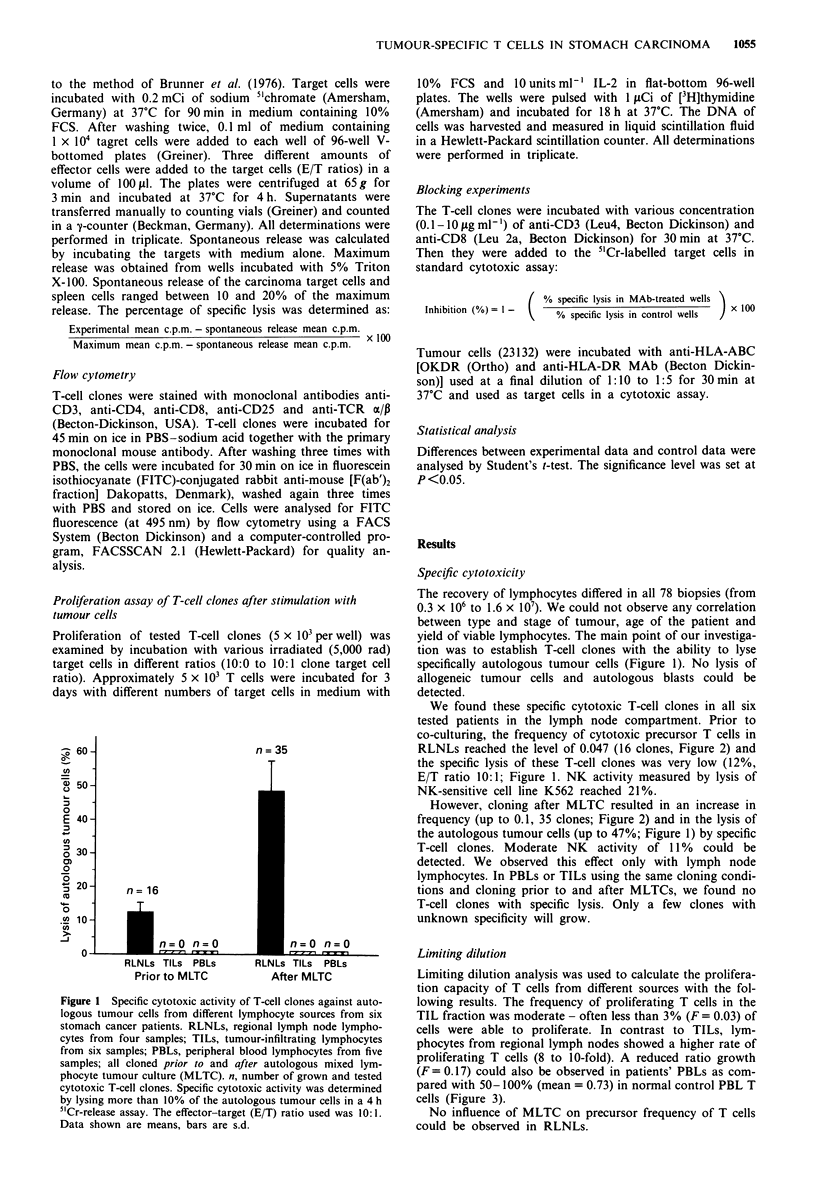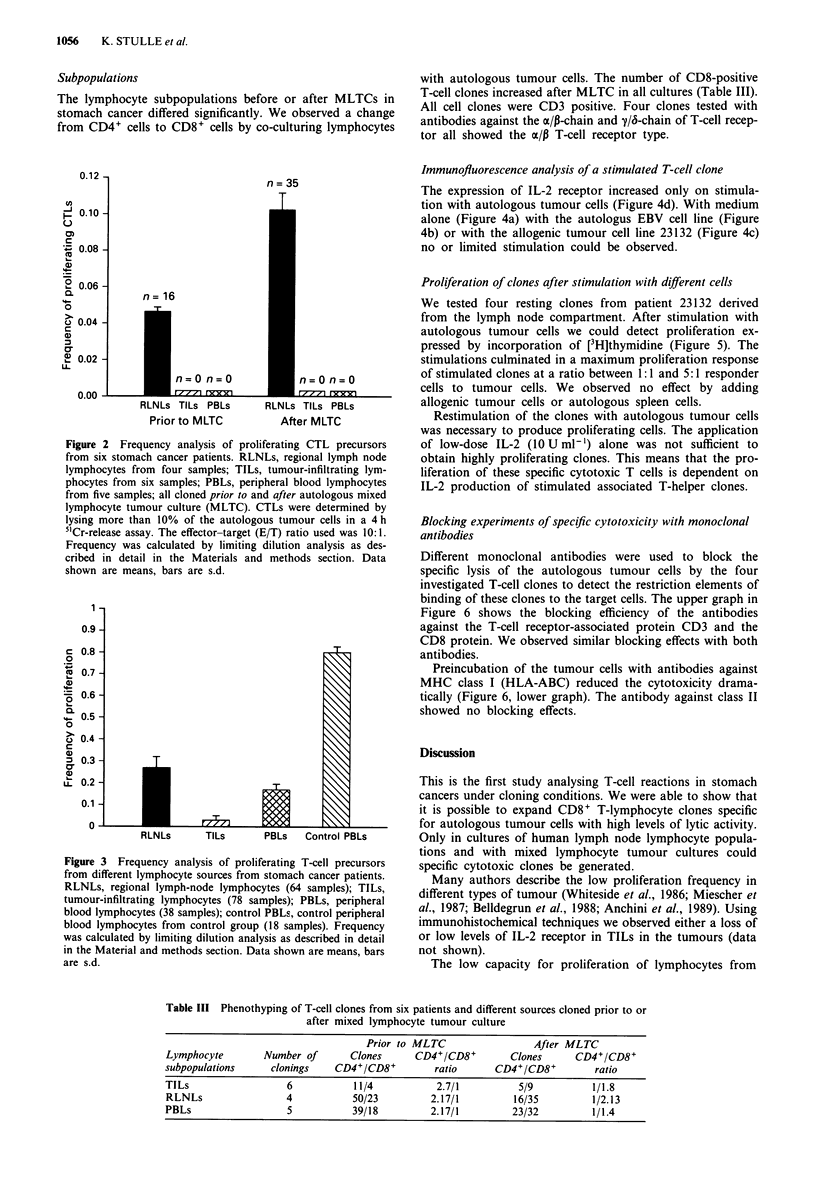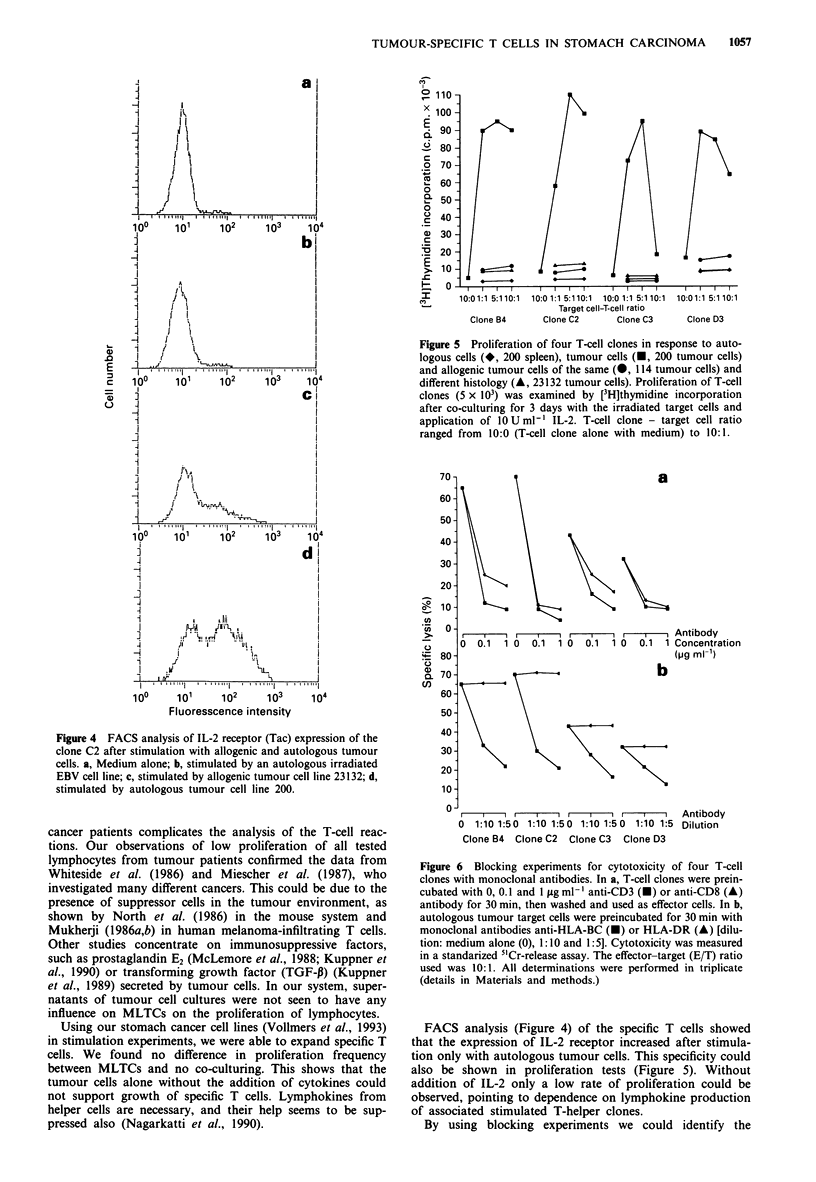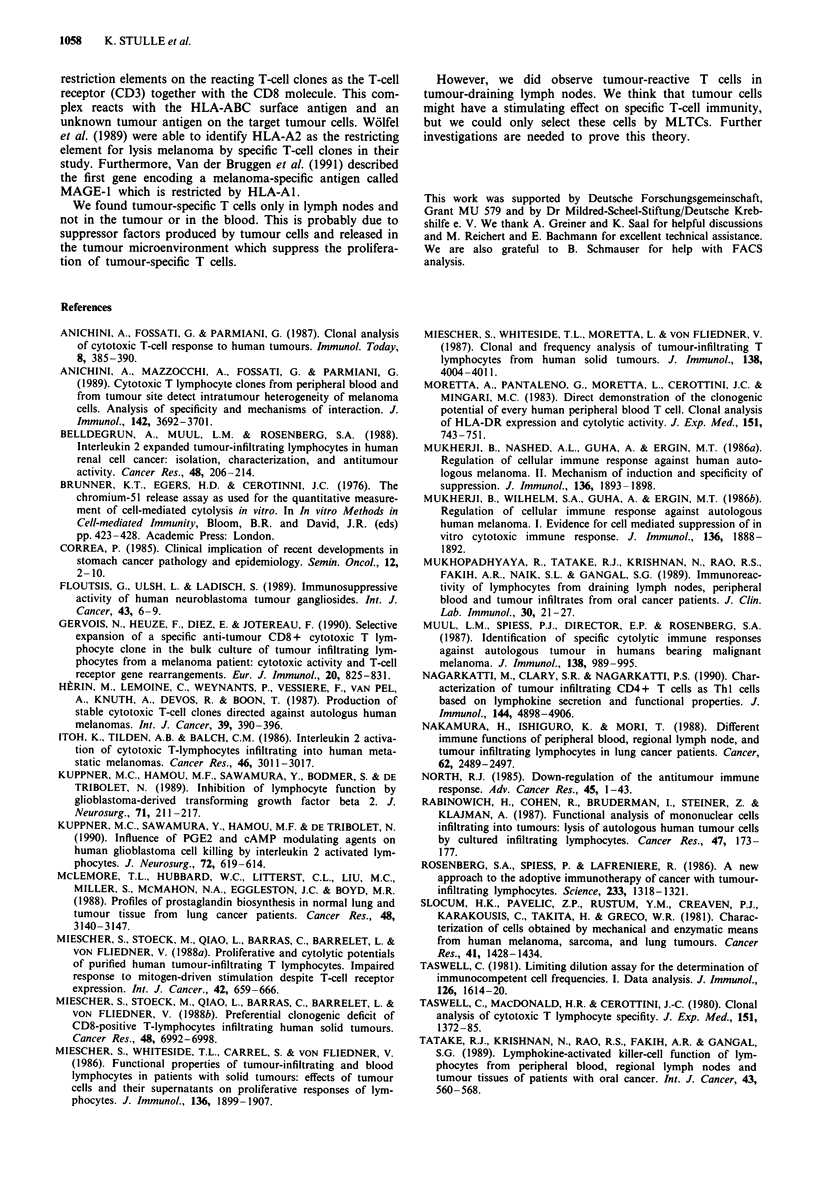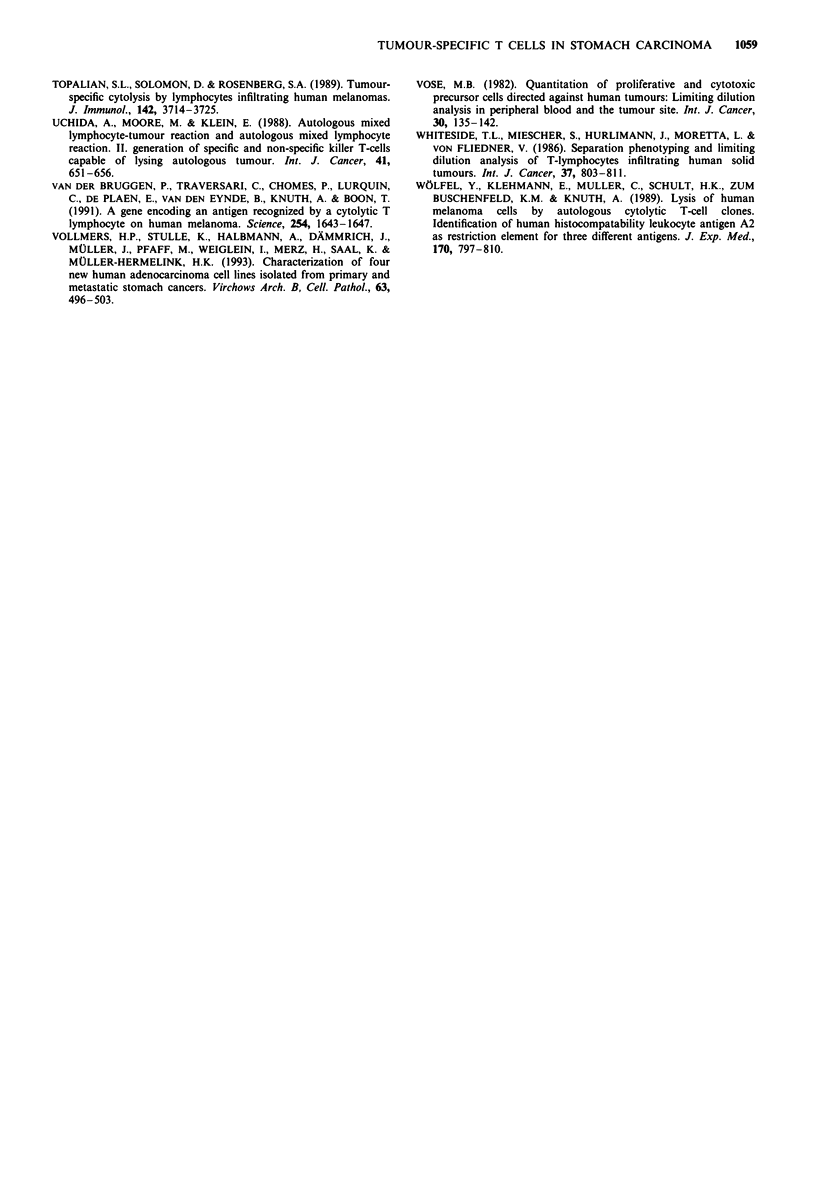# Human stomach carcinoma-specific T cells derived from the tumour-draining lymph nodes.

**DOI:** 10.1038/bjc.1994.448

**Published:** 1994-12

**Authors:** K. Stulle, H. P. Vollmers, P. Marquardt, H. K. Müller-Hermelink

**Affiliations:** Pathologisches Institut, Universität Würzburg, Germany.

## Abstract

In this paper we investigate the reactivity pattern of T cells from stomach carcinoma patients against autologous tumour cells. T cells obtained from the tumour environment, tumour-draining lymph nodes and peripheral blood were cloned in 78 patients with stomach cancer and anti-tumour cytotoxic T lymphocytes (CTLs) precursor frequencies were assessed in each sample by using limiting dilution analysis. When tumour-specific CTLs were tested for specific T-cell killing by using only low doses of Interleukin 2 (100 U ml-1), a moderate rate of proliferation frequency of T cells (0.047) and specific cytotoxicity (12%) were observed in lymph node populations. When both IL-2 and autologous tumour cells in mixed lymphocyte tumour cultures (MLTCs) were used for stimulation, a dramatic increase in number (0.1) and in specific lytic activity (46%) could be measured. No effect or specific activity to tumour cells was observed with peripheral blood lymphocytes and tumour-infiltrating lymphocytes.


Br. J. Cancer (1994), **70**, 1053–1059 © Macmillan Press Ltd., 1994

### Human stomach carcinoma-specific T cells derived from the tumour-draining lymph nodes

K. Stulle, H.P. Vollmers, P. Marquardt & H.K. Müller-Hermelink

*Pathologisches Institut, Universität Würzburg, Josef-Schneider Str. 2, 97078 Würzburg, Germany.*

**Summary** In this paper we investigate the reactivity pattern of T cells from stomach carcinoma patients against autologous tumour cells. T cells obtained from the tumour environment, tumour-draining lymph nodes and peripheral blood were cloned in 78 patients with stomach cancer and anti-tumour cytotoxic T lymphocytes (CTLs) precursor frequencies were assessed in each sample by using limiting dilution analysis. When tumour-specific CTLs were tested for specific T-cell killing by using only low doses of Interleukin 2 (100 U $ml^{-1}$), a moderate rate of proliferation frequency of T cells (0.047) and specific cytotoxicity (12%) were observed in lymph node populations. When both IL-2 and autologous tumour cells in mixed lymphocyte tumour cultures (MLTCs) were used for stimulation, a dramatic increase in number (0.1) and in specific lytic activity (46%) could be measured. No effect or specific activity to tumour cells was observed with peripheral blood lymphocytes and tumour-infiltrating lymphocytes.

Most investigations of T-cell reactivity to tumours have up until now concentrated on melanomas. This is a good model of cellular reaction to tumour-associated immunogenic structures (Anchini *et al.*, 1987, 1989; Muul *et al.*, 1987; Topalian *et al.*, 1989) and one in which immunotherapies have proved effective (Rosenberg *et al.*, 1986). Only a few other spontaneous tumours have been analysed, and only a few reports (Nakamura *et al.*, 1988; Mukhopadhyaya *et al.*, 1989; Tatake *et al.*, 1989) have compared immune responses between lymphocytes isolated from peripheral blood, regional lymph nodes and tumours in different malignant diseases. Most deal with lymphokine-activated killer (LAK) cells (Itoh *et al.*, 1986; Rabinowich *et al.*, 1987) and cytotoxic T lymphocytes (CTLs) originating from melanomas (Muul *et al.*, 1987; Anchini *et al.*, 1989; Topalian *et al.*, 1989). In many cases, it has been reported that tumours themselves (Miescher *et al.*, 1986; McLemore *et al.*, 1988; Floutsis *et al.*, 1989; Kuppner *et al.*, 1989, 1990) or tumour-infiltrating suppressor cells (North *et al.*, 1985; Mukherji *et al.*, 1986*a*,*b*) inhibit the proliferation and function of T lymphocytes. Hérin *et al.* (1987), Uchida *et al.* (1988) and Gervois *et al.* (1990) showed in their experiments that mixed lymphocyte tumour cultures (MLTCs) can propagate the growth of tumour-specific cytotoxic T cells *in vitro*.

Stomach carcinoma is still one of the most frequent cancers worldwide with a very poor prognosis (Correa, 1985) and relatively unknown aetiology. Furthermore, most likely owing to the long development of these spontaneous tumours, a down-regulation of host immune response has been observed (Whiteside *et al.*, 1986; Miescher *et al.*, 1987; Belldegrun *et al.*, 1988). The proliferation capacity of immune cells from tumour patients seems to be very poor (Miescher *et al.*, 1988*a*,*b*), making it difficult to investigate reactivity patterns of T cells from stomach cancer.

In this study we raised and analysed tumour-specific CTLs obtained from stomach carcinoma patients.

### Materials and methods

### Patients

Seventy-eight different stomach tumour resections from 78 German patients between the ages of 29 and 83 (mean 65 years) were used in our study (Table I). We obtained only one tumour from every patient. In 46 cases the clinical resections included the spleen. From 64 patients lymph nodes had been resected and from 14 we received no lymph node material. Blood was obtained from 38 patients prior to operation. The lymphocytes from one source (tumour, blood, spleen or lymph node) each tested as a separate population. None of the patients had received chemotherapy before resection.

Correspondence: K. Stulle, Pathologisches Institut, Universität Würzburg, Josef-Schneider Str. 2, 97078 Würzburg, Germany.
Received 24 June 1994; and in revised form 22 July 1994.

### Isolation of lymphocytes and tumour cells

*Peripheral blood lymphocytes (PBLs)* Heparinised blood obtained prior to the operation was diluted 1:1 with RPMI-1640 (Biochrom, Germany). Mononuclear cells were isolated by Ficoll–Hypaque (Biochrom) density gradient. A 25 ml volume of diluted blood overlayered on 15 ml of Ficoll ($D = 0.0177$) was centrifuged at 700 $g$ for 20 min at room temperature. Cells from the interphase were collected and washed twice in RPMI-1640.

*Regional lymph node lymphocytes (RLNLs)* Regional lymph nodes, harvested from resection, were minced and pressed through a 100 μm nylon mesh (Nybolt, Switzerland). The suspended cells were washed twice in 10 ml of RPMI + 10% fetal calf serum (FCS) (Biochrom) + 50 μg $ml^{-1}$ gentamycin (Biochrom).

*Tumour-infiltrating lymphocytes (TILs)* The tumour pieces from 78 tumours were minced, crushed on a glass slide, passed through a 100 μm stainless-steel mesh and washed in phosphate-buffered saline (PBS) according to the method of Vose (1982). The remaining tissue was treated with collagenase II (Sigma, Germany) and DNAse I (Sigma) according to Slocum *et al.* (1983). Cell suspensions were then

**Table I** Total number of stomach cancer cases analysed

| *Tumour type*[a] | *Number* | *Sex (male/female)* | *Mean age* | *Tumour grade*[b] *G1* | *G2* | *G3* | *G4* |
|---|---|---|---|---|---|---|---|
| Signet ring Carcinoma | 6 | 2/4 | 58.0 | – | 1 | 3 | 2 |
| Undifferentiated Carcinoma | 20 | 13/7 | 62.7 | – | 2 | 10 | 8 |
| Adenocarcinoma | 52 | 34/18 | 68.0 · | 6 | 22 | 21 | 3 |

[a]Tumour type assigned was according to the WHO histological classification of malignant tumours of the stomach. [b]Tumour grade was histopathologically determined according to cellular components, with G1 indicating a well-differentiated, G2 a moderately well-differentiated and G3–G4 a poorly to very poorly differentiated adenocarcinoma.

layered on a two-step Ficoll–Hypaque gradient (100% Ficoll and 33% Ficoll in RPMI). The cells were centrifuged for 30 min at 400 g. Tumour cells were found in the interphase of 33% Ficoll dilution. Between 20 and 87% (mean 47%, determined from May–Grünwald–Giemsa-stained cytospins) of cells harvested at the 100% Ficol interphase were lymphocytes (TILs). These TILs were resuspended in RPMI + 10% FCS for later use in limiting dilution and MLTCs.

*Tumour cell culture*

Freshly isolated tumour cells were transferred to 500 ml plastic culture flasks (Greiner, Germany) and incubated for 30 min at 37°C to remove the macrophages and majority of fibroblasts by adherence on the plastic surface. Then $0.5 \times 10^6$ non-adherent cells were cultured in a 25 ml culture flask (Greiner) in RPMI-1640 supplemented with 10% FCS and antibiotics to obtain permanent tumour cell lines. The remaining tumour cells were frozen and stored in liquid nitrogen. Immunohistological studies had shown that the tumour cells were contaminated with 10–20% remaining fibroblasts. When needed for stimulation and targets in $^{51}Cr$-release assay, tumour cells were washed and irradiated (5,000 rad).

Tumorigenicity, immunohistochemical studies and biochemical analysis of the six established tumour cell lines were as described by Vollmers et al. (1993).

*Mixed lymphocyte tumour cultures*

To select tumour specific T cells, we modified the method of Uchida et al. (1988) and Hérin et al. (1987) who used autologous tumour cells for stimulation. From one tumour (23132) and from five lymph node metastases we derived stable tumour cell lines to use in co-culturing. MLTCs were layered out from every tumour by using 24-well flat-bottomed tissue culutre plates (Greiner) with $1 \times 10^6$ responder and $1 \times 10^5$ irradiated (5,000 rad) autologous tumour cells (10:1). The cells were mixed in final volume of 2 ml of RPMI + 10% FCS and 1% gentamicin. On day 3 50 IU ml$^{-1}$ recombinant IL-2 was added. On day 7 the growing cells were collected, washed and incubated with fresh irradiated tumour cells (2 ml of medium containing 50 IU ml$^{-1}$ rIL-2). On day 14 lymphocytes were washed, counted, phenotyped by monoclonal antibody OKT3 (Ortho Diagnostics, Germany) and later used in limiting dilution experiments.

*Cell cloning by limiting dilution*

All lymphocytes were phenotyped by monoclonal antibody OKT3. Cloning by limiting dilution of T cells was performed according to the method of Moretta et al. (1982). Clones were established from fresh PBLs, RLNLs or TILs and after MLTCs. Microcultures of all lymphocytes were grown in 96-well round-bottomed microtitre plates (Greiner). From each of the 78 patients only one cloning of each different lymphoid source was performed. Four 96-well plates were seeded per cloning, one with 1, one with 3, one with 10 and one with 30 cells per well seeded in 200 µl of complete medium containing 50 IU ml$^{-1}$ rIL-2 and 1% phytohaemagglutinin (PHA) (Gibco, Germany), supplemented weekly with $1 \times 10^5$ irradiated feeder cells (allogeneic spleen cells) suspended in 100 µl of medium with IL-2 and in six cases with $1 \times 10^3$ irradiated autologous tumour cells. After 3 weeks the growing responder cells were transferred to 96-well flat-bottomed microtitre plates (200 µl of medium supplemented with 50 IU ml$^{-1}$ rIL-2, feeder cells and in six cases with $1 \times 10^3$ irradiated autologous tumour cells). Expanding microcultures were plated on 24-well tissue culture plates (Greiner), evaluated for their phenotype and functionally tested in chromium-release assay (Brunner et al., 1976) against five allogeneic tumour target cells, autologous tumour cells and PHA-blasts and K562 cells.

Owing to the low proliferative T-cell precursor frequency obtained in these samples, and according to a formula described in Taswell et al. (1980), explanded microcultures were operationally defined as clones.

*Determination of frequencies of proliferating T-cell precursors by limiting dilution analysis*

Precursor frequencies were obtained by calculating and drawing a straight line which describes the relationship between number of seeded cells per well and the logarithm of the fraction of non-proliferating (negative) microcultures. According to the single-hit Poisson model, the cell dose ($x$-axis) containing on the average one proliferating T cell is given by the intercept of the straight titration line by the ordinate value 0.37. Positive/negative responses were the presence/absence of growing T cells in a well at the highest three dilutions. Results were calculated using a program established and described in more detail by Taswell (1981).

*Culture of T-cell clones*

Obtained clones were cultured in RPMI with 10% FCS and antibiotics supplemented with 50 IU ml$^{-1}$ rIL-2. The addition of irradiated (5,000 rad) autologous tumour cells every 2 weeks in an effector–target (E/T) ratio of 10:1 was necessary to maintain growth of the specific clones.

*Target cells*

Six different stomach carcinoma cell lines, the natural killer cell (NK)-sensitive cell line K562 and the NK-resistant cell line Daudi were used as allogenic target cells in cytotoxic assay and MLTC (Table II). All lines were obtained from metastatic stomach tumours after isolation and culturing as described above. PHA-blasts were generated by stimulation of freshly isolated autologous lymphocytes from blood or spleen in medium supplemented with 1% PHA (Gibco) for 3 days, washed and used as target cells after labelling with $^{51}Cr$.

*Cytotoxic assay*

The cytotoxic activity of T-cell clones was tested for cytotoxic activity against $^{51}Cr$-labelled target cells according

**Table II** Tumour cell lines used in mixed lymphocyte tumour culture

| Number of tumour | Sex of patient | Age of patient | Tumour type[a] | Tumour stage[b] | Tumour grade[c] | Tumour cell origin |
|---|---|---|---|---|---|---|
| 114 | Female | 54 | Signet ring | T4 N1 M1 | G3 | Primary tumour |
| 200 | Female | 44 | Signet ring | T4 N1 M0 | G4 | Primary tumour |
| 2474 | Male | 60 | Adenocarcinoma | T1 N2 M1 | G2 | Metastasis |
| 2957 | Male | 51 | Adenocarcinoma | T2 N2 M0 | G3 | Metastasis |
| 3051 | Male | 62 | Adenocarcinoma | T4 N2 M0 | G3 | Metastasis |
| 23132 | Male | 72 | Adenocarcinoma | T2 N0 M0 | G3 | Primary tumour |

[a]Tumour type was assigned according to the WHO histological classification of malignant tumours of the stomach. [b]Tumour stage proposed by the American Joint Committee on Cancer, based on the TNM classification. [c]Tumour grade was histopathologically determined according to cellular components, with G1 indicating a well-differentiated, G2 a moderately well-differentiated and G3–G4 a poorly to very poorly differentiated adenocarcinoma.

to the method of Brunner *et al.* (1976). Target cells were incubated with 0.2 mCi of sodium $^{51}$chromate (Amersham, Germany) at 37°C for 90 min in medium containing 10% FCS. After washing twice, 0.1 ml of medium containing $1 \times 10^4$ tagret cells were added to each well of 96-well V-bottomed plates (Greiner). Three different amounts of effector cells were added to the target cells (E/T ratios) in a volume of 100 µl. The plates were centrifuged at 65 *g* for 3 min and incubated at 37°C for 4 h. Supernatants were transferred manually to counting vials (Greiner) and counted in a γ-counter (Beckman, Germany). All determinations were performed in triplicate. Spontaneous release was calculated by incubating the targets with medium alone. Maximum release was obtained from wells incubated with 5% Triton X-100. Spontaneous release of the carcinoma target cells and spleen cells ranged between 10 and 20% of the maximum release. The percentage of specific lysis was determined as:

$$\frac{\text{Experimental mean c.p.m.} - \text{spontaneous release mean c.p.m.}}{\text{Maximum mean c.p.m.} - \text{spontaneous release mean c.p.m.}} \times 100$$

*Flow cytometry*

T-cell clones were stained with monoclonal antibodies anti-CD3, anti-CD4, anti-CD8, anti-CD25 and anti-TCR α/β (Becton-Dickinson, USA). T-cell clones were incubated for 45 min on ice in PBS–sodium acid together with the primary monoclonal mouse antibody. After washing three times with PBS, the cells were incubated for 30 min on ice in fluorescein isothiocyanate (FITC)-conjugated rabbit anti-mouse [F(ab')$_2$ fraction] Dakopatts, Denmark), washed again three times with PBS and stored on ice. Cells were analysed for FITC fluorescence (at 495 nm) by flow cytometry using a FACS System (Becton Dickinson) and a computer-controlled program, FACSSCAN 2.1 (Hewlett-Packard) for quality analysis.

*Proliferation assay of T-cell clones after stimulation with tumour cells*

Proliferation of tested T-cell clones ($5 \times 10^3$ per well) was examined by incubation with various irradiated (5,000 rad) target cells in different ratios (10:0 to 10:1 clone target cell ratio). Approximately $5 \times 10^3$ T cells were incubated for 3 days with different numbers of target cells in medium with 10% FCS and 10 units ml$^{-1}$ IL-2 in flat-bottom 96-well plates. The wells were pulsed with 1 µCi of [$^3$H]thymidine (Amersham) and incubated for 18 h at 37°C. The DNA of cells was harvested and measured in liquid scintillation fluid in a Hewlett-Packard scintillation counter. All determinations were performed in triplicate.

*Blocking experiments*

The T-cell clones were incubated with various concentration (0.1–10 µg ml$^{-1}$) of anti-CD3 (Leu4, Becton Dickinson) and anti-CD8 (Leu 2a, Becton Dickinson) for 30 min at 37°C. Then they were added to the $^{51}$Cr-labelled target cells in standard cytotoxic assay:

$$\text{Inhibition (\%)} = 1 - \left( \frac{\text{\% specific lysis in MAb-treated wells}}{\text{\% specific lysis in control wells}} \right) \times 100$$

Tumour cells (23132) were incubated with anti-HLA-ABC [OKDR (Ortho) and anti-HLA-DR MAb (Becton Dickinson)] used at a final dilution of 1:10 to 1:5 for 30 min at 37°C and used as target cells in a cytotoxic assay.

*Statistical analysis*

Differences between experimental data and control data were analysed by Student's *t*-test. The significance level was set at $P<0.05$.

### Results

*Specific cytotoxicity*

The recovery of lymphocytes differed in all 78 biopsies (from $0.3 \times 10^6$ to $1.6 \times 10^7$). We could not observe any correlation between type and stage of tumour, age of the patient and yield of viable lymphocytes. The main point of our investigation was to establish T-cell clones with the ability to lyse specifically autologous tumour cells (Figure 1). No lysis of allogeneic tumour cells and autologous blasts could be detected.

We found these specific cytotoxic T-cell clones in all six tested patients in the lymph node compartment. Prior to co-culturing, the frequency of cytotoxic precursor T cells in RLNLs reached the level of 0.047 (16 clones, Figure 2) and the specific lysis of these T-cell clones was very low (12%, E/T ratio 10:1; Figure 1. NK activity measured by lysis of NK-sensitive cell line K562 reached 21%.

However, cloning after MLTC resulted in an increase in frequency (up to 0.1, 35 clones; Figure 2) and in the lysis of the autologous tumour cells (up to 47%; Figure 1) by specific T-cell clones. Moderate NK activity of 11% could be detected. We observed this effect only with lymph node lymphocytes. In PBLs or TILs using the same cloning conditions and cloning prior to and after MLTCs, we found no T-cell clones with specific lysis. Only a few clones with unknown specificity will grow.

*Limiting dilution*

Limiting dilution analysis was used to calculate the proliferation capacity of T cells from different sources with the following results. The frequency of proliferating T cells in the TIL fraction was moderate – often less than 3% ($F = 0.03$) of cells were able to proliferate. In contrast to TILs, lymphocytes from regional lymph nodes showed a higher rate of proliferating T cells (8 to 10-fold). A reduced ratio growth ($F = 0.17$) could also be observed in patients' PBLs as compared with 50–100% (mean = 0.73) in normal control PBL T cells (Figure 3).

No influence of MLTC on precursor frequency of T cells could be observed in RLNLs.

**Figure 1** Specific cytotoxic activity of T-cell clones against autologous tumour cells from different lymphocyte sources from six stomach cancer patients. RLNLs, regional lymph node lymphocytes from four samples; TILs, tumour-infiltrating lymphocytes from six samples; PBLs, peripheral blood lymphocytes from five samples; all cloned *prior to* and *after* autologous mixed lymphocyte tumour culture (MLTC). *n*, number of grown and tested cytotoxic T-cell clones. Specific cytotoxic activity was determined by lysing more than 10% of the autologous tumour cells in a 4 h $^{51}$Cr-release assay. The effector–target (E/T) ratio used was 10:1. Data shown are means, bars are s.d.

*Subpopulations*

The lymphocyte subpopulations before or after MLTCs in stomach cancer differed significantly. We observed a change from $CD4^+$ cells to $CD8^+$ cells by co-culturing lymphocytes with autologous tumour cells. The number of CD8-positive T-cell clones increased after MLTC in all cultures (Table III). All cell clones were CD3 positive. Four clones tested with antibodies against the α/β-chain and γ/δ-chain of T-cell receptor all showed the α/β T-cell receptor type.

**Figure 2** Frequency analysis of proliferating CTL precursors from six stomach cancer patients. RLNLs, regional lymph node lymphocytes from four samples; TILs, tumour-infiltrating lymphocytes from six samples; PBLs, peripheral blood lymphocytes from five samples; all cloned *prior to* and *after* autologous mixed lymphocyte tumour culture (MLTC). CTLs were determined by lysing more than 10% of the autologous tumour cells in a 4 h $^{51}$Cr-release assay. The effector–target (E/T) ratio used was 10:1. Frequency was calculated by limiting dilution analysis as described in detail in the Materials and methods section. Data shown are means, bars are s.d.

**Figure 3** Frequency analysis of proliferating T-cell precursors from different lymphocyte sources from stomach cancer patients. RLNLs, regional lymph-node lymphocytes (64 samples); TILs, tumour-infiltrating lymphocytes (78 samples); PBLs, peripheral blood lymphocytes (38 samples); control PBLs, control peripheral blood lymphocytes from control group (18 samples). Frequency was calculated by limiting dilution analysis as described in detail in the Material and methods section. Data shown are means, bars are s.d.

*Immunofluorescence analysis of a stimulated T-cell clone*

The expression of IL-2 receptor increased only on stimulation with autologous tumour cells (Figure 4d). With medium alone (Figure 4a) with the autologus EBV cell line (Figure 4b) or with the allogenic tumour cell line 23132 (Figure 4c) no or limited stimulation could be observed.

*Proliferation of clones after stimulation with different cells*

We tested four resting clones from patient 23132 derived from the lymph node compartment. After stimulation with autologous tumour cells we could detect proliferation expressed by incorporation of [$^3$H]thymidine (Figure 5). The stimulations culminated in a maximum proliferation response of stimulated clones at a ratio between 1:1 and 5:1 responder cells to tumour cells. We observed no effect by adding allogenic tumour cells or autologous spleen cells.

Restimulation of the clones with autologous tumour cells was necessary to produce proliferating cells. The application of low-dose IL-2 (10 U $ml^{-1}$) alone was not sufficient to obtain highly proliferating clones. This means that the proliferation of these specific cytotoxic T cells is dependent on IL-2 production of stimulated associated T-helper clones.

*Blocking experiments of specific cytotoxicity with monoclonal antibodies*

Different monoclonal antibodies were used to block the specific lysis of the autologous tumour cells by the four investigated T-cell clones to detect the restriction elements of binding of these clones to the target cells. The upper graph in Figure 6 shows the blocking efficiency of the antibodies against the T-cell receptor-associated protein CD3 and the CD8 protein. We observed similar blocking effects with both antibodies.

Preincubation of the tumour cells with antibodies against MHC class I (HLA-ABC) reduced the cytotoxicity dramatically (Figure 6, lower graph). The antibody against class II showed no blocking effects.

### Discussion

This is the first study analysing T-cell reactions in stomach cancers under cloning conditions. We were able to show that it is possible to expand $CD8^+$ T-lymphocyte clones specific for autologous tumour cells with high levels of lytic activity. Only in cultures of human lymph node lymphocyte populations and with mixed lymphocyte tumour cultures could specific cytotoxic clones be generated.

Many authors describe the low proliferation frequency in different types of tumour (Whiteside *et al.*, 1986; Miescher *et al.*, 1987; Belldegrun *et al.*, 1988; Anchini *et al.*, 1989). Using immunohistochemical techniques we observed either a loss of or low levels of IL-2 receptor in TILs in the tumours (data not shown).

The low capacity for proliferation of lymphocytes from

**Table III** Phenothyping of T-cell clones from six patients and different sources cloned prior to or after mixed lymphocyte tumour culture

| *Lymphocyte subpopulations* | *Number of clonings* | *Prior to MLTC* Clones $CD4^+/CD8^+$ | *Prior to MLTC* $CD4^+/CD8^+$ ratio | *After MLTC* Clones $CD4^+/CD8^+$ | *After MLTC* $CD4^+/CD8^+$ ratio |
|---|---|---|---|---|---|
| TILs | 6 | 11/4 | 2.7/1 | 5/9 | 1/1.8 |
| RLNLs | 4 | 50/23 | 2.17/1 | 16/35 | 1/2.13 |
| PBLs | 5 | 39/18 | 2.17/1 | 23/32 | 1/1.4 |

**Figure 4** FACS analysis of IL-2 receptor (Tac) expression of the clone C2 after stimulation with allogenic and autologous tumour cells. **a**, Medium alone; **b**, stimulated by an autologous irradiated EBV cell line; **c**, stimulated by allogenic tumour cell line 23132; **d**, stimulated by autologous tumour cell line 200.

**Figure 5** Proliferation of four T-cell clones in response to autologous cells (◆, 200 spleen), tumour cells (■, 200 tumour cells) and allogenic tumour cells of the same (●, 114 tumour cells) and different histology (▲, 23132 tumour cells). Proliferation of T-cell clones ($5 \times 10^3$) was examined by [$^3$H]thymidine incorporation after co-culturing for 3 days with the irradiated target cells and application of $10\,U\,ml^{-1}$ IL-2. T-cell clone – target cell ratio ranged from 10:0 (T-cell clone alone with medium) to 10:1.

**Figure 6** Blocking experiments for cytotoxicity of four T-cell clones with monoclonal antibodies. In **a**, T-cell clones were preincubated with 0, 0.1 and $1\,\mu g\,ml^{-1}$ anti-CD3 (■) or anti-CD8 (▲) antibody for 30 min, then washed and used as effector cells. In **b**, autologous tumour target cells were preincubated for 30 min with monoclonal antibodies anti-HLA-BC (■) or HLA-DR (▲) [dilution: medium alone (0), 1:10 and 1:5]. Cytotoxicity was measured in a standarized $^{51}$Cr-release assay. The effector–target (E/T) ratio used was 10:1. All determinations were performed in triplicate (details in Materials and methods.)

cancer patients complicates the analysis of the T-cell reactions. Our observations of low proliferation of all tested lymphocytes from tumour patients confirmed the data from Whiteside *et al.* (1986) and Miescher *et al.* (1987), who investigated many different cancers. This could be due to the presence of suppressor cells in the tumour environment, as shown by North *et al.* (1986) in the mouse system and Mukherji (1986*a*,*b*) in human melanoma-infiltrating T cells. Other studies concentrate on immunosuppressive factors, such as prostaglandin $E_2$ (McLemore *et al.*, 1988; Kuppner *et al.*, 1990) or transforming growth factor (TGF-$\beta$) (Kuppner *et al.*, 1989) secreted by tumour cells. In our system, supernatants of tumour cell cultures were not seen to have any influence on MLTCs on the proliferation of lymphocytes.

Using our stomach cancer cell lines (Vollmers *et al.*, 1993) in stimulation experiments, we were able to expand specific T cells. We found no difference in proliferation frequency between MLTCs and no co-culturing. This shows that the tumour cells alone without the addition of cytokines could not support growth of specific T cells. Lymphokines from helper cells are necessary, and their help seems to be suppressed also (Nagarkatti *et al.*, 1990).

FACS analysis (Figure 4) of the specific T cells showed that the expression of IL-2 receptor increased after stimulation only with autologous tumour cells. This specificity could also be shown in proliferation tests (Figure 5). Without addition of IL-2 only a low rate of proliferation could be observed, pointing to dependence on lymphokine production of associated stimulated T-helper clones.

By using blocking experiments we could identify the restriction elements on the reacting T-cell clones as the T-cell receptor (CD3) together with the CD8 molecule. This complex reacts with the HLA-ABC surface antigen and an unknown tumour antigen on the target tumour cells. Wölfel *et al.* (1989) were able to identify HLA-A2 as the restricting element for lysis melanoma by specific T-cell clones in their study. Furthermore, Van der Bruggen *et al.* (1991) described the first gene encoding a melanoma-specific antigen called MAGE-1 which is restricted by HLA-A1.

We found tumour-specific T cells only in lymph nodes and not in the tumour or in the blood. This is probably due to suppressor factors produced by tumour cells and released in the tumour microenvironment which suppress the proliferation of tumour-specific T cells.

However, we did observe tumour-reactive T cells in tumour-draining lymph nodes. We think that tumour cells might have a stimulating effect on specific T-cell immunity, but we could only select these cells by MLTCs. Further investigations are needed to prove this theory.

This work was supported by Deutsche Forschungsgemeinschaft, Grant MU 579 and by Dr Mildred-Scheel-Stiftung/Deutsche Krebshilfe e. V. We thank A. Greiner and K. Saal for helpful discussions and M. Reichert and E. Bachmann for excellent technical assistance. We are also grateful to B. Schmauser for help with FACS analysis.

### References

ANICHINI, A., FOSSATI, G. & PARMIANI, G. (1987). Clonal analysis of cytotoxic T-cell response to human tumours. *Immunol. Today*, **8**, 385–390.

ANICHINI, A., MAZZOCCHI, A., FOSSATI, G. & PARMIANI, G. (1989). Cytotoxic T lymphocyte clones from peripheral blood and from tumour site detect intratumour heterogeneity of melanoma cells. Analysis of specificity and mechanisms of interaction. *J. Immunol.*, **142**, 3692–3701.

BELLDEGRUN, A., MUUL, L.M. & ROSENBERG, S.A. (1988). Interleukin 2 expanded tumour-infiltrating lymphocytes in human renal cell cancer: isolation, characterization, and antitumour activity. *Cancer Res.*, **48**, 206–214.

BRUNNER, K.T., EGERS, H.D. & CEROTINNI, J.C. (1976). The chromium-51 release assay as used for the quantitative measurement of cell-mediated cytolysis *in vitro*. In *In vitro Methods in Cell-mediated Immunity*, Bloom, B.R. and David, J.R. (eds) pp. 423–428. Academic Press: London.

CORREA, P. (1985). Clinical implication of recent developments in stomach cancer pathology and epidemiology. *Semin. Oncol.*, **12**, 2–10.

FLOUTSIS, G., ULSH, L. & LADISCH, S. (1989). Immunosuppressive activity of human neuroblastoma tumour gangliosides. *Int. J. Cancer*, **43**, 6–9.

GERVOIS, N., HEUZE, F., DIEZ, E. & JOTEREAU, F. (1990). Selective expansion of a specific anti-tumour CD8+ cytotoxic T lymphocyte clone in the bulk culture of tumour infiltrating lymphocytes from a melanoma patient: cytotoxic activity and T-cell receptor gene rearrangements. *Eur. J. Immunol.*, **20**, 825–831.

HÈRIN, M., LEMOINE, C., WEYNANTS, P., VESSIERE, F., VAN PEL, A., KNUTH, A., DEVOS, R. & BOON, T. (1987). Production of stable cytotoxic T-cell clones directed against autologus human melanomas. *Int. J. Cancer*, **39**, 390–396.

ITOH, K., TILDEN, A.B. & BALCH, C.M. (1986). Interleukin 2 activation of cytotoxic T-lymphocytes infiltrating into human metastatic melanomas. *Cancer Res.*, **46**, 3011–3017.

KUPPNER, M.C., HAMOU, M.F., SAWAMURA, Y., BODMER, S. & DE TRIBOLET, N. (1989). Inhibition of lymphocyte function by glioblastoma-derived transforming growth factor beta 2. *J. Neurosurg.*, **71**, 211–217.

KUPPNER, M.C., SAWAMURA, Y., HAMOU, M.F. & DE TRIBOLET, N. (1990). Influence of PGE2 and cAMP modulating agents on human glioblastoma cell killing by interleukin 2 activated lymphocytes. *J. Neurosurg.*, **72**, 619–614.

MCLEMORE, T.L., HUBBARD, W.C., LITTERST, C.L., LIU, M.C., MILLER, S., MCMAHON, N.A., EGGLESTON, J.C. & BOYD, M.R. (1988). Profiles of prostaglandin biosynthesis in normal lung and tumour tissue from lung cancer patients. *Cancer Res.*, **48**, 3140–3147.

MIESCHER, S., STOECK, M., QIAO, L., BARRAS, C., BARRELET, L. & VON FLIEDNER, V. (1988*a*). Proliferative and cytolytic potentials of purified human tumour-infiltrating T lymphocytes. Impaired response to mitogen-driven stimulation despite T-cell receptor expression. *Int. J. Cancer.*, **42**, 659–666.

MIESCHER, S., STOECK, M., QIAO, L., BARRAS, C., BARRELET, L. & VON FLIEDNER, V. (1988*b*). Preferential clonogenic deficit of CD8-positive T-lymphocytes infiltrating human solid tumours. *Cancer Res.*, **48**, 6992–6998.

MIESCHER, S., WHITESIDE, T.L., CARREL, S. & VON FLIEDNER, V. (1986). Functional properties of tumour-infiltrating and blood lymphocytes in patients with solid tumours: effects of tumour cells and their supernatants on proliferative responses of lymphocytes. *J. Immunol.*, **136**, 1899–1907.

MIESCHER, S., WHITESIDE, T.L., MORETTA, L. & VON FLIEDNER, V. (1987). Clonal and frequency analysis of tumour-infiltrating T lymphocytes from human solid tumours. *J. Immunol.*, **138**, 4004–4011.

MORETTA, A., PANTALENO, G., MORETTA, L., CEROTTINI, J.C. & MINGARI, M.C. (1983). Direct demonstration of the clonogenic potential of every human peripheral blood T cell. Clonal analysis of HLA-DR expression and cytolytic activity. *J. Exp. Med.*, **151**, 743–751.

MUKHERJI, B., NASHED, A.L., GUHA, A. & ERGIN, M.T. (1986*a*). Regulation of cellular immune response against human autologous melanoma. II. Mechanism of induction and specificity of suppression. *J. Immunol.*, **136**, 1893–1898.

MUKHERJI, B., WILHELM, S.A., GUHA, A. & ERGIN, M.T. (1986*b*). Regulation of cellular immune response against autologous human melanoma. I. Evidence for cell mediated suppression of in vitro cytotoxic immune response. *J. Immunol.*, **136**, 1888–1892.

MUKHOPADHYAYA, R., TATAKE, R.J., KRISHNAN, N., RAO, R.S., FAKIH, A.R., NAIK, S.L. & GANGAL, S.G. (1989). Immunoreactivity of lymphocytes from draining lymph nodes, peripheral blood and tumour infiltrates from oral cancer patients. *J. Clin. Lab. Immunol.*, **30**, 21–27.

MUUL, L.M., SPIESS, P.J., DIRECTOR, E.P. & ROSENBERG, S.A. (1987). Identification of specific cytolytic immune responses against autologous tumour in humans bearing malignant melanoma. *J. Immunol.*, **138**, 989–995.

NAGARKATTI, M., CLARY, S.R. & NAGARKATTI, P.S. (1990). Characterization of tumour infiltrating CD4+ T cells as Th1 cells based on lymphokine secretion and functional properties. *J. Immunol.*, **144**, 4898–4906.

NAKAMURA, H., ISHIGURO, K. & MORI, T. (1988). Different immune functions of peripheral blood, regional lymph node, and tumour infiltrating lymphocytes in lung cancer patients. *Cancer*, **62**, 2489–2497.

NORTH, R.J. (1985). Down-regulation of the antitumour immune response. *Adv. Cancer Res.*, **45**, 1–43.

RABINOWICH, H., COHEN, R., BRUDERMAN, I., STEINER, Z. & KLAJMAN, A. (1987). Functional analysis of mononuclear cells infiltrating into tumours: lysis of autologous human tumour cells by cultured infiltrating lymphocytes. *Cancer Res.*, **47**, 173–177.

ROSENBERG, S.A., SPIESS, P. & LAFRENIERE, R. (1986). A new approach to the adoptive immunotherapy of cancer with tumour-infiltrating lymphocytes. *Science*, **233**, 1318–1321.

SLOCUM, H.K., PAVELIC, Z.P., RUSTUM, Y.M., CREAVEN, P.J., KARAKOUSIS, C., TAKITA, H. & GRECO, W.R. (1981). Characterization of cells obtained by mechanical and enzymatic means from human melanoma, sarcoma, and lung tumours. *Cancer Res.*, **41**, 1428–1434.

TASWELL, C. (1981). Limiting dilution assay for the determination of immunocompetent cell frequencies. I. Data analysis. *J. Immunol.*, **126**, 1614–20.

TASWELL, C., MACDONALD, H.R. & CEROTTINI, J.-C. (1980). Clonal analysis of cytotoxic T lymphocyte specifity. *J. Exp. Med.*, **151**, 1372–85.

TATAKE, R.J., KRISHNAN, N., RAO, R.S., FAKIH, A.R. & GANGAL, S.G. (1989). Lymphokine-activated killer-cell function of lymphocytes from peripheral blood, regional lymph nodes and tumour tissues of patients with oral cancer. *Int. J. Cancer*, **43**, 560–568.

TOPALIAN, S.L., SOLOMON, D. & ROSENBERG, S.A. (1989). Tumour-specific cytolysis by lymphocytes infiltrating human melanomas. *J. Immunol.*, **142**, 3714–3725.

UCHIDA, A., MOORE, M. & KLEIN, E. (1988). Autologous mixed lymphocyte-tumour reaction and autologous mixed lymphocyte reaction. II. generation of specific and non-specific killer T-cells capable of lysing autologous tumour. *Int. J. Cancer*, **41**, 651–656.

VAN DER BRUGGEN, P., TRAVERSARI, C., CHOMES, P., LURQUIN, C., DE PLAEN, E., VAN DEN EYNDE, B., KNUTH, A. & BOON, T. (1991). A gene encoding an antigen recognized by a cytolytic T lymphocyte on human melanoma. *Science*, **254**, 1643–1647.

VOLLMERS, H.P., STULLE, K., HALBMANN, A., DÄMMRICH, J., MÜLLER, J., PFAFF, M., WEIGLEIN, I., MERZ, H., SAAL, K. & MÜLLER-HERMELINK, H.K. (1993). Characterization of four new human adenocarcinoma cell lines isolated from primary and metastatic stomach cancers. *Virchows Arch. B, Cell. Pathol.*, **63**, 496–503.

VOSE, M.B. (1982). Quantitation of proliferative and cytotoxic precursor cells directed against human tumours: Limiting dilution analysis in peripheral blood and the tumour site. *Int. J. Cancer*, **30**, 135–142.

WHITESIDE, T.L., MIESCHER, S., HURLIMANN, J., MORETTA, L. & VON FLIEDNER, V. (1986). Separation phenotyping and limiting dilution analysis of T-lymphocytes infiltrating human solid tumours. *Int. J. Cancer*, **37**, 803–811.

WÖLFEL, Y., KLEHMANN, E., MULLER, C., SCHULT, H.K., ZUM BUSCHENFELD, K.M. & KNUTH, A. (1989). Lysis of human melanoma cells by autologous cytolytic T-cell clones. Identification of human histocompatability leukocyte antigen A2 as restriction element for three different antigens. *J. Exp. Med.*, **170**, 797–810.